# The Contemporary Role of Speckle Tracking Echocardiography in Cirrhotic Cardiomyopathy

**DOI:** 10.3390/life14020179

**Published:** 2024-01-25

**Authors:** Yannis Dimitroglou, Constantina Aggeli, Alexandra Alexopoulou, Dimitrios Tsartsalis, Dimitrios Patsourakos, Markos Koukos, Dimitris Tousoulis, Konstantinos Tsioufis

**Affiliations:** 1 First Department of Cardiology, Medical School, National and Kapodistrian University of Athens, Hippokration General Hospital, 115 27 Athens, Greece; kaggeli@med.uoa.gr (C.A.); dtsartsalis@gmail.com (D.T.); dpatsourakos@yahoo.com (D.P.); marckoukos@yahoo.gr (M.K.); ktsioufis@hippocratio.gr (K.T.); 2 Second Department of Medicine & Research Laboratory, Medical School, National and Kapodistrian University of Athens, Hippokration General Hospital, 115 27 Athens, Greece; alexopou@ath.forthnet.gr

**Keywords:** liver cirrhosis, cirrhotic cardiomyopathy, speckle tracking echocardiography, global longitudinal strain

## Abstract

Cirrhotic cardiomyopathy (CCM) is characterized by elevated cardiac output at rest, an inability to further increase contractility under stress, and diastolic dysfunction. The diagnosis of CCM is crucial as it can lead to complications during liver transplantation. However, its recognition poses challenges with conventional echocardiography techniques. Speckle tracking echocardiography (STE), particularly global longitudinal strain (GLS), is a novel index that enhances the diagnostic efficacy of echocardiography for both ischemic and non-ischemic cardiomyopathies. GLS proves more sensitive in identifying early systolic dysfunction and is also influenced by advanced diastolic dysfunction. Consequently, there is an expanding scope for GLS utilization in cirrhotic cases, with newly updated diagnostic criteria for CCM incorporating GLS. Specifically, systolic dysfunction is now defined as either a left ventricular ejection fraction below 50% or an absolute GLS below 18%. However, conflicting data on GLS alterations in liver cirrhosis patients persist, as many individuals with advanced disease and a poor prognosis exhibit a hyperdynamic state with preserved or increased GLS. Consequently, the presence of CCM, according to the updated criteria, does not exhibit a significant association—in the majority of studies—with the severity of liver disease and prognosis. Furthermore, information on other indices measured with STE, such as left atrial and right ventricular strain, is promising but currently limited. This review aims to offer a critical assessment of the existing evidence concerning the application of STE in patients with liver cirrhosis.

## 1. Introduction

The term cirrhotic cardiomyopathy (CCM) encompasses alterations in cardiovascular function observed in patients with liver cirrhosis (LC), particularly those with end-stage liver disease [1]. CCM is marked by a high cardiac output state and reduced afterload at rest, combined with a compromised ability to increase contractility under stress and diastolic dysfunction (DD) [2]. Cardiac dysfunction is often masked due to the reduced afterload, which diminishes cardiac work [3]. Nevertheless, exposure to acute events or procedures such as transjugular intrahepatic portosystemic shunt (TIPS) and liver transplantation (LT) can unveil the underlying cardiac impairment [4]. Studies report that up to 21% of post-LT fatalities are attributable to heart failure [5]. Accordingly, the European Association for the Study of the Liver (EASL) guidelines mandate transthoracic echocardiography for all LT candidates, with stress echocardiography recommended in select cases for preprocedural evaluation and risk stratification [6].

Despite these recommendations, CCM often eludes detection through conventional cardiovascular imaging modalities. Consequently, more sensitive echocardiography indices, such as speckle tracking echocardiography (STE), have emerged as valuable tools for identifying subclinical myocardial dysfunction in cirrhotic patients [7]. Among these indices, Global Longitudinal Strain (GLS) stands out as a sensitive parameter capable of revealing early abnormalities in patients with otherwise preserved systolic function of the left ventricle (LV), as seen in LC patients [8,9]. GLS is incorporated into the modified criteria for CCM, specifically defining systolic dysfunction [10]. Nevertheless, the available data on GLS and other STE-measured parameters in cirrhotic patients are currently limited.

This review aims to provide a critical evaluation of the current evidence regarding the use of STE in patients with LC.

## 2. Pathophysiology of CCM

Cardiac dysfunction in patients with LC is the result of the cardiac remodeling that ensues as a compensatory response to the high output state [7]. The high output state is precipitated by low peripheral resistances induced by arterial vasodilation in the splanchnic circulation. Indeed, alterations in systemic circulation have been correlated with changes in hepatic blood flow and portal pressure [11]. Underlying mechanisms include the dysfunction of liver sinusoidal endothelial cells, which increases hepatic vascular resistance, triggering signals that increase the production of vascular endothelial growth factor, angiotensin-(1–7), endocannabinoids, nitric oxide synthase, carbon monoxide, adrenomedullin, and prostacyclin, among others [12,13,14,15]. These molecules facilitate the formation of collateral portosystemic vessels and induce peripheral vasodilation [16]. Despite the activation of the sympathetic nervous system, vascular smooth muscle cells fail to constrict properly in response to adrenergic stimulation [17].

The increased cardiac output results not only from high stroke volume but also from an elevated resting heart rate [18]. This is accompanied by an initial decrease in effective arterial blood volume due to vasodilation, prompting renin production. Activation of the renin–angiotensin–aldosterone system further augments the total intravascular volume [19]. These changes are accompanied by desensitization of b-adrenergic receptors and inflammation-induced alterations in the myocardium, leading to a blunted response to stress, a hallmark of CCM [20,21].

**Figure 1:**
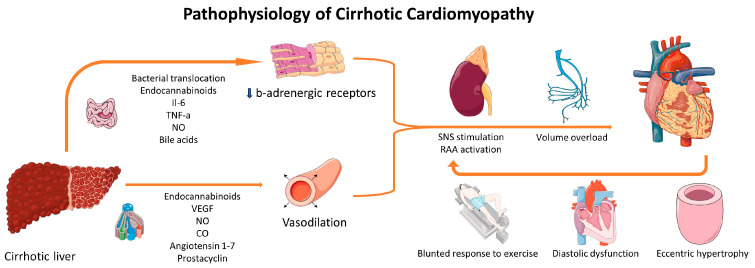
Pathophysiological mechanisms implicated in CCM. Several molecules produced by the cirrhotic liver or by the induced inflammation result in vasodilation and desensitization of b-adrenergic receptors. These changes result in RAA activation and adrenergic stimulation, which are responsible for volume overload, eccentric cardiac hypertrophy, DD, and a blunted response to stress. The resulting cardiac dysfunction can, in turn, induce further activation of RAAS and SNS. Parts of the figure were drawn using pictures from Servier Medical Art (https://creativecommons.org/licenses/by/3.0/, 21 November 2023). CO: carbon monoxide; IL-6: interleukin-6; NO: nitric oxide; RAAS: renin-angiotensin-aldosterone system; SNS: sympathetic nervous system; VEGF: vascular endothelial growth factor.

In patients with LC, the high output state induces LV remodeling characterized by increased ventricular dimensions, augmented LV mass, and expanded left atrial (LA) volume, irrespective of etiology [22]. Autopsy studies have confirmed increased LV dimensions and mass, particularly in patients with ascites and alcoholic etiology [23]. Left ventricular hypertrophy, possibly accompanied by edema and fibrosis as indicated by cardiac magnetic resonance studies, can lead to DD, another hallmark of CCM [24]. DD may result in increased end-diastolic LV pressure, further elevating LA volume, and has been associated with lower quality of life and worse survival in certain studies, though not consistently across all literature [25,26]. Figure 1 summarizes pathophysiological mechanisms implicated in the emergence of CCM.

Advanced echocardiography, particularly STE, has enhanced diagnostic efficacy in identifying subtle systolic and DD [27]. Nonetheless, the debate persists on whether these advancements contribute to a more accurate and clinically or prognostically significant diagnosis of CCM.

## 3. Diagnosis of CCM

According to the Montreal Criteria, introduced in 2005, CCM was characterized by the presence of systolic or diastolic dysfunction, along with additional indicators such as increased left ventricular mass, QTc interval abnormalities, elevated natriuretic peptide values, and an abnormal response to stress [2]. However, ongoing advancements in cardiovascular imaging, particularly tissue Doppler imaging (TDI), and the updated guidelines from the American Society of Echocardiography (ASE) and European Association of Cardiovascular Imaging (EACVI) for DD, alongside the emergence of STE, have prompted the formulation of revised CCM criteria [10]. According to the redefined criteria, CCM is diagnosed when either systolic or diastolic dysfunction is detected in an echocardiography study at rest. Systolic dysfunction is defined as a left ventricular ejection fraction (LVEF) <50% or an absolute GLS value below 18%. The initial criteria included patients with GLS higher than 22%, but this was later withdrawn. [10] DD is diagnosed according to the ASE/EACVI guidelines slightly modified for patients with LC. Table 1 presents traditional and redefined criteria for the diagnosis of CCM.

The prevalence of DD was relatively high under the 2005 criteria, potentially leading to overdiagnosis of CCM. A systematic review published in 2019, which assessed DD based on previous criteria, found its presence in 51.2% of cirrhotic patients [28]. However, according to the updated guidelines, DD occurs in substantially fewer patients [29,30]. Marella et al., who evaluated the diastolic function of 400 consecutive cirrhotic patients based on the newer diastolic dysfunction criteria, confirmed DD in only 50 out of 266 patients with sufficient images and measurements (18.7%) [31]. As DD is less prevalent in the redefined criteria, the systolic element for CCM diagnosis could potentially identify CCM in more patients. However, given that most patients with LC exhibit preserved LVEF at rest, a comprehensive analysis of data regarding STE and GLS is warranted.

## 4. How Different Are Left Ventricular Strain Values of Cirrhotic Patients When Compared to Controls?

Initial studies that have aimed to assess the systolic function of the left ventricle using strain parameters utilized the systolic element of Tissue Doppler Imaging (TDI). In one such study, strain and strain rate were measured in forty-four cirrhotic patients, revealing worse parameters than those in controls [32]. However, a more recent study with a larger cohort, employing a similar methodology to measure strain, did not confirm these findings [18].

Most initial studies implementing STE reported lower absolute values of GLS in patients with cirrhosis compared to controls. Sampaio et al. conducted a case-control study using apical four and two-chamber views to calculate peak systolic longitudinal strain in 109 patients and 18 controls. Peak systolic longitudinal strain was lower in patients than in controls (19.99% vs. 22.02% respectively, *p* = 0.003) [33]. Notably, peak systolic strain was calculated as the mean peak strain of each myocardial segment and is not identical to GLS, which is calculated at end-systole. Altekin et al. calculated GLS in 38 cirrhotics and 37 controls, reporting significantly lower absolute GLS in patients compared to controls (20.6% vs. 28.7%, respectively, *p* < 0.001) [34]. Although the reported value for the patient group falls within normal limits, the mean value for the control group is above the normal range, as reported elsewhere. Chen et al. also reported lower absolute GLS values in patients with LC compared to controls (18.6% vs. 20.1%, respectively, *p* < 0.01) [35]. They also observed an improvement in GLS after liver transplantation. Regarding other strain parameters besides longitudinal strain, Pagourelias et al. reported non-different circumferential strain and improved radial strain in cirrhotic patients [36]. On the contrary, Inci et al. reported worse radial strain in the patient group [37]. Another study reported similar values in these measurements [38]. To the best of our knowledge, other novel STE parameters, such as tissue mitral annular displacement, have not been studied in cirrhotics [39].

Studies published later, when guidelines for GLS measurement were more standardized, yielded varying results. Most studies did not find statistically significant differences between patients and controls [38,40,41], while some studies found lower absolute values for patients with cirrhosis than for controls [37,42]. Some studies even found increased GLS values in cirrhotic patients compared to controls. Kim et al. reported substantially higher absolute GLS in cirrhotics awaiting liver transplantation than in controls (24.2% vs. 18.6%, *p* < 0.001) [43]. Von Köckritz et al. measured GLS for the endocardium, mid-myocardium, and epicardium, finding higher absolute GLS values for all three distinct values and their average (21.4% vs. 18.7%, *p* < 0.001) [44]. Similar results were also found by Zamirian et al., who reported that while absolute GLS was higher in cirrhotic patients, it increased to a greater degree in controls than in patients during a dobutamine stress test [45].

Data concerning GLS in patients with liver cirrhosis were summarized in a recently published meta-analysis by Ridjab et al., including 20 cross-sectional, case-control, or cohort studies. In 19 of these studies reporting GLS values, a total of 1017 patients and 609 controls were included. The investigators found lower absolute GLS values in patients with liver cirrhosis than in controls [Mean Difference −1.43% (−2.79%–0.07%), *p* = 0.04] [46]. However, this analysis reported increased heterogeneity between studies (I^2^ = 0.95, *p* < 0.00001), did not include all relevant studies [36,47], including a study in non-cirrhotic patients and namely those with chronic hepatitis B [48], and incorporated a study that calculated longitudinal strain with cardiac magnetic resonance [49]. Therefore, further well-designed studies are needed before drawing conclusions on how GLS values in patients with LC differentiate from the normal range and how the etiology of LC may affect those values.

## 5. Are Left Ventricular Strain Values Associated with Disease Severity and Prognosis?

Most studies utilizing speckle tracking analysis in cirrhotic patients have relatively small sample sizes; hence, only a few present subgroup analyses or prognostic data. Association with disease severity was performed by either measuring GLS across the Child-Pugh classes (class A for compensated disease, class B for intermediate disease severity, and class C for very advanced decompensated disease) or by correlating GLS with the Model for End-stage Liver Disease (MELD). The latter is calculated from creatinine, bilirubin, and international normalized ratio values and has been shown to be very well and significantly associated with the prognosis of liver disease [50,51,52]. Pagourelias et al. measured speckle tracking parameters in 77 cirrhotic patients and found that longitudinal strain did not differ across the three Child-Pugh classes (20.1%, 21.3%, 21.0% for Child Pugh A, B, and C, respectively) [36]. Anish et al. also did not report significant differences between patients who had Model for End-stage Liver Disease (MELD) scores below and above 12 (−19.76% and −19.25%, respectively) [53]. Dimitroglou et al. found a significant association between GLS and disease severity. They found that the absolute GLS value was significantly higher in Child-Pugh B and C patients (22.2% and 23.0%, respectively) compared to Child Pugh A patients (20.3%). They also found a significant association between GLS and MELD in multivariate models [54]. Skouloudi et al. demonstrated that patients with MELD scores above 15 had significantly higher absolute GLS compared to patients with MELD scores below 15. This finding was also evident in the multivariate analysis, where the MELD score was the only factor associated with GLS [55]. Mechelink et al. found that patients with decompensated LC had increased absolute GLS values compared to patients with compensated LC without portal hypertension. They also found that GLS values in patients with compensated LC but signs of portal hypertension had higher GLS compared to those without portal hypertension and were similar to those with decompensated LC. When a dobutamine stress echo was performed, GLS values were similar in the three groups [56].

Several studies have also sought to investigate the potential association between GLS and patient prognosis. A prospective study published by Sampaio et al. compared patients hospitalized for complications of LC vs. outpatients. They found that GLS (mean absolute value 19.5%) did not differ between the two groups and was not associated with an adverse prognosis [57]. Similar findings were also reported by Nazar et al., who also used four and two-chamber views for the calculation of the GLS [58]. In a retrospective study measuring echocardiographic parameters in two cohorts of patients who were candidates for liver transplantation, Jansen et al. found that increased absolute GLS was associated with reduced transplant-free survival. However, in the subgroup analysis, GLS was associated with earlier LT, which could have confounded the results. Additionally, mean GLS values were substantially lower than those found in other relevant studies [59]. In another retrospective cohort of patients undergoing TIPS, Jansen et al. found that low absolute GLS was associated with a worse prognosis [60]. With such conflicting results, we could hypothesize that even though increased absolute GLS may be seen in patients with advanced liver cirrhosis, who typically have a poor prognosis, significantly reduced values may be associated with worse outcomes, even in patients with compensated liver disease.

## 6. In What Proportion of Cirrhotic Patients Do GLS Values Satisfy the Systolic Criterion for the Diagnosis of CCM?

Several studies have aimed to quantify the proportion of cirrhotic patients who could be diagnosed with CCM based on the redefined criteria. However, the change in the systolic criterion and the withdrawal of values higher than 22% from the diagnosis have led to confusion and potential heterogeneity in the published data. Razpotnik et al. investigated the prevalence of CCM according to both criteria. The prevalence was slightly and not significantly higher according to the Montreal Criteria (67.2% vs. 55.7%, *p* = 0.09). Interestingly, the diastolic criterion was satisfied in 64.8% according to the older criteria and in 7.4% according to the redefined criteria (*p* < 0.0001). On the contrary, the systolic criterion was satisfied in 16.4% according to the older criteria and in 53.3% according to the redefined criteria (*p* < 0.001) [61]. Specifically, reduced LVEF was found in 2.5%, GLS < 18% with preserved EF in 9.8%, and GLS > 22% in 41% of patients. Cesari et al. presented that CCM could be diagnosed in 29% of cirrhotic patients. Interestingly, in 25% of the patient sample, the GLS value was abnormal based on the CCM criteria, elaborating that CCM diagnosis was made based on GLS values. It is also worth noting that GLS values did not differ between patients with CCM or without CCM and controls (21 ± 3%, 20 ± 1%, and 20 ± 1%, respectively) [62]. Dimitroglou et al. found that abnormal GLS values (either <18 or >22) were evident in 48.5% of patients, with none of them having an LVEF value below 50% [63]. Skouloudi et al., on the other hand, calculated the prevalence of CCM based on the presence of GLS <18 and found that only 3.7% of patients satisfied the systolic criterion [55]. Interestingly, all aforementioned studies reported that CCM diagnosis was not associated with the disease prognosis. These data indicate that further studies are required before implementing GLS as a strong diagnostic index of CCM. The normal range may also need further adjustments to match the special characteristics of cirrhotic patients. Table 2 summarizes data from studies having measured longitudinal strain parameters in cirrhotic patients.

## 7. Speckle Tracking Echocardiography for the Evaluation of Left Atrial Function in Cirrhotic Patients

Studies published before the development of STE have presented increased dimensions and volume of the left atrium (LA) in patients with liver cirrhosis. Specifically, Silvestre et al. found a significant association between left atrial size and MELD score, and patients with MELD above 16 had a significantly higher dimension of the LA when compared to patients with MELD below 16 (42.05 ± 5.40 mm vs. 38.96 ± 4.63 mm, *p* < 0.001) [65]. Licata et al. also presented increased left atrial volume in patients when compared to control and in patients with ascites when compared to patients without ascites [66]. Regarding the association with the prognosis of the disease, Cesari et al. presented that the left atrial dimension was associated with a worse prognosis when the MELD score was not included in the models. However, the inclusion of MELD blunted the prognostic significance of the left atrial dimension [67].

Regarding strain measurements, data are scarce but provide insightful information. Von Kochritz et al. reported that LA reservoir and conduit strain, as well as LA strain rate, were significantly reduced in patients with advanced cirrhosis when compared to controls. They also reported a trend toward reduced post-transplant survival in patients with a reduced LA reservoir and conduit strain [44]. Dimitroglou et al. did not find a significant association between LA reservoir strain and MELD score but found an association with the E/e’ ratio, indicating an association of LA strain with DD [68]. Similarly, Meucci et al. reported that patients with advanced DD grade had worse values of LA strain and, interestingly, worse prognosis. Specifically, Kaplan-Meyer analysis showed that patients with LA strain ≤ 35% had an increased event rate when compared to patients with LA strain <35% (58% vs. 29%, Log-rank test: *p* = 0.001) [64]. Skouloudi et al. did not find an association between LA strain and MELD score. However, in the prospective study, they presented that patients with reduced LA reservoir strain had a worse prognosis in the multivariate model, which also included MELD (HR 0.96, 95%CI 0.93–0.99; *p*  =  0.017) [55]. These initial findings are consistent in that LA strain does not differ so profoundly between patients and controls, as the left atrial dimension indicates. It could, however, be a more specific marker of cardiac dysfunction and worse prognosis than the traditional echocardiographic indices used for the evaluation of the LA.

## 8. Speckle Tracking Echocardiography for the Evaluation of Right Ventricular and Right Atrial Function in Cirrhotic Patients

Right cardiac chambers may also be affected in patients with LC. Autopsy studies have shown that right ventricular (RV) hypertrophy and dilatation are more prevalent in such patients [69]. Echocardiography studies have indicated that increased right ventricular afterload, as estimated by pulmonary artery systolic pressure and right atrial volume, is associated with a worse prognosis [70]. RV strain in cardiovascular disease is a useful tool for the evaluation of the RV function. Specifically, worse RV strain is associated with worse survival both in pressure overload conditions, such as in pulmonary hypertension, and in volume overload conditions, such as in patients with severe tricuspid regurgitation [71,72]. It is also associated with a worse prognosis in patients with left-sided heart failure [73]. However, the implementation of STE in right cardiac chambers has been shown to have limitations in the past, and therefore, only a few such studies have been published in patients with LC.

Chen et al. presented that patients with liver cirrhosis have lower RV total strain than controls [35]. However, in their analysis, they did not include RV-free wall strain, which has been shown to be more reliable for the evaluation of RV function [74]. Zhang et al. found that RV-free wall strain is lower than in controls and is associated with bilirubin but not with MELD score. They also presented worse values of right atrial reservoir strain in patients than in controls [75]. In contrast to those findings, Rimbas et al. did not find significant differences in RV global longitudinal strain between patients and controls [38]. Skouloudi et al. presented that RV-free wall strain did not differ between patients with MELD lower or above 15 and that it was not associated with a worse prognosis [55].

## 9. Conclusions

Cirrhotic cardiomyopathy is characterized by subtle myocardial damage shown to have prognostic significance, especially in disease-modifying procedures, such as TIPS and LT. Its recognition, though with conventional echocardiography, is challenging, and therefore, several studies have examined the potential role of STE, and particularly GLS, as a diagnostic and prognostic tool. In addition, GLS has been introduced into the diagnostic criteria for cirrhotic cardiomyopathy, but emerging data have shown that the association between GLS and liver disease severity and prognosis may not be as straightforward as initially anticipated. Therefore, further multicenter prospective studies are required to guide the appropriate implementation of STE for the diagnosis of cirrhotic cardiomyopathy.

## Figures and Tables

**Table 1:** Traditional and redefined criteria for the diagnosis of CCM.

2005 Montreal Criteria	
Systolic criteria (at least one)	Blunted increase in cardiac output with exercise, volume challenge or pharmacologic stimuli LVEF at rest < 55%
Diastolic criteria (at least one)	Ε/A < 1 Ε wave deceleration time > 200 ms Isovolumetric relaxation time > 80 ms
Supportive data	Blunted chronotropic response to stress Electromechanical uncoupling Dyssynchrony QT prolongation LA dilatation Increased LV mass Increased BNP or proBNP Increased troponin Ι
**2019 Cirrhotic Cardiomyopathy Consortium Criteria**	
Systolic criteria (at least one)	LVEF < 50% GLS < 18
Diastolic criteria (more than 50%)	Septal e’ < 7 cm/s E/e’ > 15 LAVi > 34 mL/m^2^ TR velocity > 2.8 m/s

BNP: brain natriuretic peptide; GLS: global longitudinal strain; LA: left atrial; LAVi: left atrial volume index; LV: left ventricular; LVEF: left ventricular ejection fraction; TR: tricuspid regurgitation.

**Table 2:** Main studies measuring longitudinal strain in patients with Cirrhosis.

Study	Published	Study Sample	Software	Main Findings	Study Limitations
Sampaio et al. [33]	2013	109 patients, 18 controls	VVI	GLS lower in patients 19.99% vs. 22.02%, *p* = 0.003	Only apical 4 and 2 chamber view used
Nazar et al. [58]	2014	50 patients	EchoPAC	GLS not associated with the grade DD or prognosis	Only apical 4 and 2 chamber views used No control group
Altekin et al. [34]	2014	38 patient, 37 controls	EchoPAC	GLS lower in patients 20.6% vs. 28.75, *p* < 0.001 GLS lower for MELD > 10.5 19.4% vs. 21.7%, *p* < 0.001	Value for patients within normal limits, value in controls supranormal Patients with alcoholic cirrhosis excluded
Pagourelias et al. [36]	2015	77 patients and 20 controls	EchoPAC	GLS higher in patients 20.9% vs. 19.0% *p* = 0.03	Only male patients included
Chen et al. [35]	2016	103 patients, 48 controls	EchoPAC	GLS lower in patients 18.6% vs. 20.1%, *p* < 0.01 GLS improvement with LT	More than 60% of patients had viral etiology
Jansen et al. [59]	2018	168 patients	TomTec	GLS lower than reference values Higher GLS associated with worse outcome	no control group, GLS associated with earlier LT may be a confounder
Rimbas et al. [38]	2018	46 patients, 46 controls	EchoPac	GLS non-different between patients and controls 20.9% vs. 20.7%, *p* > 0.05	No patients with NASH cirrhosis included Percentage of Child-Pugh C patients low
Huang et al. [40]	2019	80 patients, 29 controls	EchoPAC	GLS non-different between patients and controls 21.5% vs. 20.2%, *p* > 0.05 GLS lower in alcoholic than in HBV cirrhosis	No patients with NASH included Patients with hypotension, common in CCM excluded from the study
Inci et al. [37]	2019	40 patients, 26 controls	VVI	GLS lower in patients 16.0% vs. 17.6%, *p* = 0.002	Only 4 and 2 chamber apical views used
Zamirian et al. [45]	2019	20 patients and 10 controls	Not stated	GLS higher in patients 22.6% vs. 19.2%, *p* < 0.001 Inability to improve GLS with dobutamine	Low sample size, no detailed description of the methodology
Jansen et al. [60]	2019	114 patients	TomTEC	Lower GLS associated with worse prognosis after TIPS	Retrospective study, many echo studies excluded for low quality lack of control group
Kim et al. [43]	2020	33 patients and 17 controls	TomTec	GLS higher in patients 24.2% vs. 18.6%, *p* < 0.001	Low sample size
Mechelinck et al. [56]	2020	117 patients	TomTec	GLS higher in patients with decompensated LC or portal hypertension	Retrospective study No control group
Köckritz et al. [44]	2021	80 patients and 30 controls	EchoPAC	GLS higher in patients 21.4% vs. 18.7, *p* < 0.001	Patients with abnormal LVEF excluded Study included only patients referred for LT
Cesari et al. [62]	2021	83 patients and 46 controls	EchoPAC	GLS non-different between patients and controls 20% vs. 20%, *p* > 0.05	Retrospective study
Soulaidopoulos et al. [47]	2021	130 patients	EchoPAC	Patients with hepatopulmonary syndrome had lower GLS	No control group included
Poojary et al. [41]	2022	70 patients and 60 controls	EchoPAC	GLS non-different between patients and controls 22.4% vs. 21.8% GLS higher for MELD >15.5	Only Child Pugh A and B patients included
Razpotnik et al. [61]	2022	122 patients	EchoPAC	GLS < 18% in 10% of patients GLS > 22% in 41% of patients	No control group
Meucci et al. [64]	2022	129 patients	EchoPAC	15% of patients had GLS < 18% GLS not associated with prognosis	No control group Study included only patients referred for TIPS
Dimitroglou et al. [63]	2023	72 patients and 18 controls	QLAB	Higher GLS associated with increased disease severity	Data for control group not presented
Skouloudi [55]	2023	135 patients	QLAB	Higher GLS associated with higher MELD score	No control group
Cao et al. [42]	2023	90 patients and 30 controls	EchoPAC	GLS lower in patients Lower GLS associated with increased disease severity	Retrospective study Only patients with HBV cirrhosis included

For consistency among studies, strain parameters are presented as absolute values. CCM: cirrhotic cardiomyopathy; GLS: global longitudinal strain; HBV: hepatitis B virus; LVEF: left ventricular ejection fraction; LT: liver transplantation, MELD: model for end-stage liver disease; PLS: peak longitudinal strain; TIPS: transjugular intrahepatic portosystemic shunt.

## Data Availability

Not applicable.
